# Sex differences in associations between maternal deprivation and alterations in hippocampal calcium-binding proteins and cognitive functions in rats

**DOI:** 10.1186/s12993-018-0142-y

**Published:** 2018-05-15

**Authors:** Hongyu Xu, Yuqin Ye, Yelu Hao, Fei Shi, Zhiqiang Yan, Guohao Yuan, Yuefan Yang, Zhou Fei, Xiaosheng He

**Affiliations:** 1 Department of Neurosurgery, Xijing Hospital, Fourth Military Medical University, Xi’an, China; 2 Faculty of Space and Aviation, Fourth Military Medicine University, Xi’an, China; 3 Department of Neurosurgery, Second Affiliated Hospital of Hunan Normal University, Changsha, China

**Keywords:** Maternal deprivation, Hippocampus, Calretinin, Calbindin-D28k, Cognitive functions, MWM test

## Abstract

**Background and objective:**

Adverse early-life experiences have been suggested as one of the key contributors to neurodevelopmental disorders, such that these experiences influence brain development, cognitive ability and mental health. Previous studies indicated that hippocampal levels of the calcium-binding proteins calretinin (CALR) and calbindin-D28k (CALB) changed in response to maternal deprivation (MD), a model for adverse early-life experiences. We investigated the effects of MD on hippocampal CALR and CALB protein levels and cognitive behaviors, and explored whether these effects were sex-related.

**Methods:**

From postnatal day 2 (PND-2) to PND-14, rat pups in the MD group were separated from their mothers for 3 h/day for comparison with pups raised normally (control). To determine hippocampal CALR and CALB levels, fluorescent immunostaining of hippocampal sections and Western blot analysis of hippocampal tissues were employed at various timepoints (PND-21, -25, -30, -35 and -40). Behavioral and cognitive changes were determined by open field test (PND-21) and Morris water maze (PND-25).

**Results:**

Western blot analysis showed changes in the hippocampal CALR and CALB levels in both male and female MD groups, compared with controls. The open field test showed reduced exploration only in male MD groups but not female MD groups. The Morris water maze tests indicated that MD caused spatial memory impairment both in male and female rats, but there was a sex difference in CALR and CALB levels.

**Conclusions:**

Male rats are relatively more vulnerable to MD stress than female rats, but both male and female rats demonstrate spatial learning impairment after exposure to MD stress. Sex difference in CALR and CALB levels may reveal the different mechanisms behind the behavioral observations.

## Background

Neurodevelopmental disorders refer to disorders of cognitive, motor, emotional and memory functions that originated from atypical development of the brain, and affect a large percentage of the population worldwide [1–3]. The most common neurodevelopmental disorders are attention-deficit/hyperactivity disorder (ADHD) and autism spectrum disorder (ASD), which mostly occur during the early years of life [4, 5]. Epidemiology studies from different countries have shown that the incidence and prevalence of ADHD and ASD has increased during recent decades [6–10].

Early life experiences have a profound influence on the development of the central nervous system (CNS) and endocrine system, and have been suggested as a key contributor to neurodevelopmental disorders [11, 12]. Adverse life events during early life have been shown to lead to changes in the hypothalamic–pituitary–adrenal (HPA) axis and cause high-anxiety-like behavior and memory impairment [12–15]. The effects can even persist throughout life [16, 17].

An important early life experience is mother-infant interaction. The quality of caregiving by the mother is a key regulator of the HPA axis neuroplasticity in the neonatal period, and is therefore very important for brain maturation and development [18–22]. Long separation from the mother, or maternal deprivation (MD), is a potent stressor during postnatal development. MD is detrimental to stress resistance [13, 22] and can result in permanent deficits [23, 24]. MD of experimental animals is now widely used as a model for postnatal stress. Examples of behavioral changes associated with MD that can persist into adulthood include anxiety, personality disorders, schizophrenia, depression, anhedonia, and memory deficits [25–28]. Previous studies have also shown that male and female rats respond differently to MD [28, 29].

The hippocampus is involved in learning and memory, behavioral adaption, and regulation of the HPA axis system, and is particularly sensitive to stress [30]. We and others have shown that MD caused functional changes of the hippocampus, and alterations in the hippocampal levels of various proteins such as calretinin (CALR) and calbindin-D28k (CALB) [31, 32]. CALR and CALB are calcium-binding proteins that are widely present in the central nervous system, function to modulate intracellular calcium concentration and neuronal excitability [33, 34], and are important for neurogenesis and neuronal survival. Studies with transgenic mice have shown that CALR and CALB also have a role in neuronal firing and regulate synaptic plasticity [33, 35, 36]. Of particular note here is that hippocampal levels of CALR and CALB are affected by MD in both sexes, but males and females may respond differently to MD stress [32]. However, the sex-specific effect of MD on cognitive functions, and the related roles of hippocampal CALR and CALB are not fully understood.

In the present study, we utilized MD as a paradigm of early-life stress to study the response of brain functions to early adverse events, and investigated the sex-specific changes in hippocampal protein levels and cognitive functions related to stress in early life.

## Methods

### Ethics statement and animal handling

All experimental procedures were performed in accordance with the Guidelines for the Care and Use of Laboratory Animals of the European Communities Council Directive of 24 November 1986 (86/609/EEC) and were approved by the Ethics Committee of Fourth Military Medical University (Approval ID XJYYLL-2015251).

Pregnant Sprague–Dawley rats were obtained from the Animal Breeding and Research Center of the Fourth Military Medical University (Xi’an, China) and housed at 21 ± 1 °C, 55 ± 5% humidity, and 12-h light/dark cycle in single cages (40 × 25 × 20 cm). The pregnant rats were checked for litters daily. Postnatal day zero (PND-0) was defined as the day of birth. All pups were weaned on PND-21 and subsequently housed in sex-matched groups of 8 rats per cage.

### Maternal deprivation procedure

From PND-2 to PND-14, rat pups of each litter (50 in total, 25 males and 25 females) in the MD group were separated from their home cages (and mothers) at 9:00 a.m. and maintained for 3 h in a single box. Electric heating plates were used to compensate for the loss of the mother’s body heat (32 ± 1 °C). No food or water was provided during the separation time. They were returned to their mother immediately at noon the same day. Rats in the control group (48 in total, 24 males and 24 females) were left undisturbed with their mother until PND-21.

### Open field test

On PND-21, six rats from each group (the male MD group, the male control group, the female MD group, and the female control group) were randomly chosen for the open field test. The arena we used is rectangular, and made of wood. The wall height is 40 cm. To minimize background stress, we transported the rats to the testing room ≥ 1 h prior to testing to allow the rats to acclimate to the experimental room. Each trial took 5 min. After each session, the apparatus was cleaned with 40% ethanol to remove the smell left by the previous rat. All data were recorded with a computerized tracking system (Ethovision 1.90, Noldus IT, Netherlands).

### Morris water maze test

On PND-25, eight rats from each group (the male MD group, the male control group, the female MD group, and the female control group) were randomly chosen for the Morris water maze (MWM) test. The apparatus is consisted of a circular water tank (160 cm in diameter and 45 cm in height), containing water (22 ± 2 °C) to a depth of 30 cm, which was rendered opaque by adding black food dye. A platform (9 cm diameter, 29 cm height) was submerged 1 cm below the water surface and placed at the midpoint of one quadrant. The swim-route was recorded by a video camera connected to a video analysis system (DigBeh-MR, Shanghai Auspicious Software Technology, China). Each rat underwent four trials per day for five consecutive days. The time durations for escape latency (interval required for the rat to find the submerged escape platform), platform crossing, and swim speed were recorded.

### Immunofluorescence

On PND-21, four rats that were randomly chosen from each group (the male MD group, the male control group, the female MD group, and the female control group), were deeply anesthetized with a pentobarbital solution (50 mg/kg/w, Sigma, USA). Transcardial perfusion was performed with a saline solution, and then with 4% paraformaldehyde (in 0.1 M sodium phosphate buffer, pH 7.4). The brains were post-fixed overnight, cryoprotected, frozen at − 80 °C, and sectioned with a cryostat in series (10 μm thick). Before blocking, free-floating sections were first treated with 1% H_2_O_2_ for 15 min and washed 3× with phosphate buffered saline. After incubation in blocking buffer (0.3% Triton X-100/5% normal serum in phosphate buffered saline) for 1 h, the sections were placed in primary antibody solution overnight at 4 °C. The following primary antibodies were used in series: rabbit anti-CALR antibody (1: 200, Millipore), and rabbit anti-CALB antibody (1: 200, Millipore). The secondary antibody was goat anti-rabbit (1:500, Santa Cruz). Sections were analyzed with a fluorescence microscope (DP70, Olympus, Japan).

### Western blot

We used western blot on PND-21, -25, -30, -35 and -40 in the MD and control rats to determine hippocampal levels of CALR and CALB. Hippocampal tissues were homogenized in buffer containing 50 mM Tris, 150 mM NaCl, 0.1% sodium dodecyl sulfate (SDS), 1% NP-40, 0.5% sodium deoxycholate (pH 8.0) and a protease inhibitor cocktail. This was centrifuged at 10 000×*g* for 10 min at 4 °C. The protein concentrations were determined with a Bio-Rad BCA protein assay kit (Pierce, USA). Protein samples were mixed with 5× SDS-PAGE sample buffer and boiled for 5 min. The proteins in the tissue samples (20 or 30 μg protein) were resolved via 14% SDS-PAGE and then transferred onto Millipore-Immobilon-P membranes (Millipore, Bedford, MA, USA).

After blocking with 5% nonfat milk in 10 mM Tris and 100 mM NaCl for 1 h at room temperature, the membranes were incubated overnight at 4 °C serially with rabbit anti-CALR antibody (1: 1500; Millipore), rabbit anti-CALB antibody (1: 1500; Millipore), and rabbit anti-actin antibody (1: 1000; Sigma). The next day, the membranes were washed 3× and then incubated with a horseradish peroxidase-linked secondary anti-rabbit antibody (1:5000, Santa Cruz, USA). After washing 3×, the membranes were visualized with an enhanced chemiluminescence Western blot system (Amersham, Arlington Height, 500 mL). The intensity of the immunoreactive bands was captured using an image analysis system (Fotodyne, Harland, WI, USA). The optical density of the bands was quantified using a Gel-PRO analyzer program (version 4.0).

### Statistical analysis

All data were analyzed using SPSS 15.0 software. One-way or 2-way analysis of variance (ANOVA) followed by post hoc analysis (Bonferroni correction) was conducted for multiple comparison with repeated measurements. The graphs were plotted using GraphPad Prism 5.0. Difference were considered significant at *p* < 0.05.

## Results

### Hippocampal CALR from PND-21 to PND-35

**Fig. 1:**
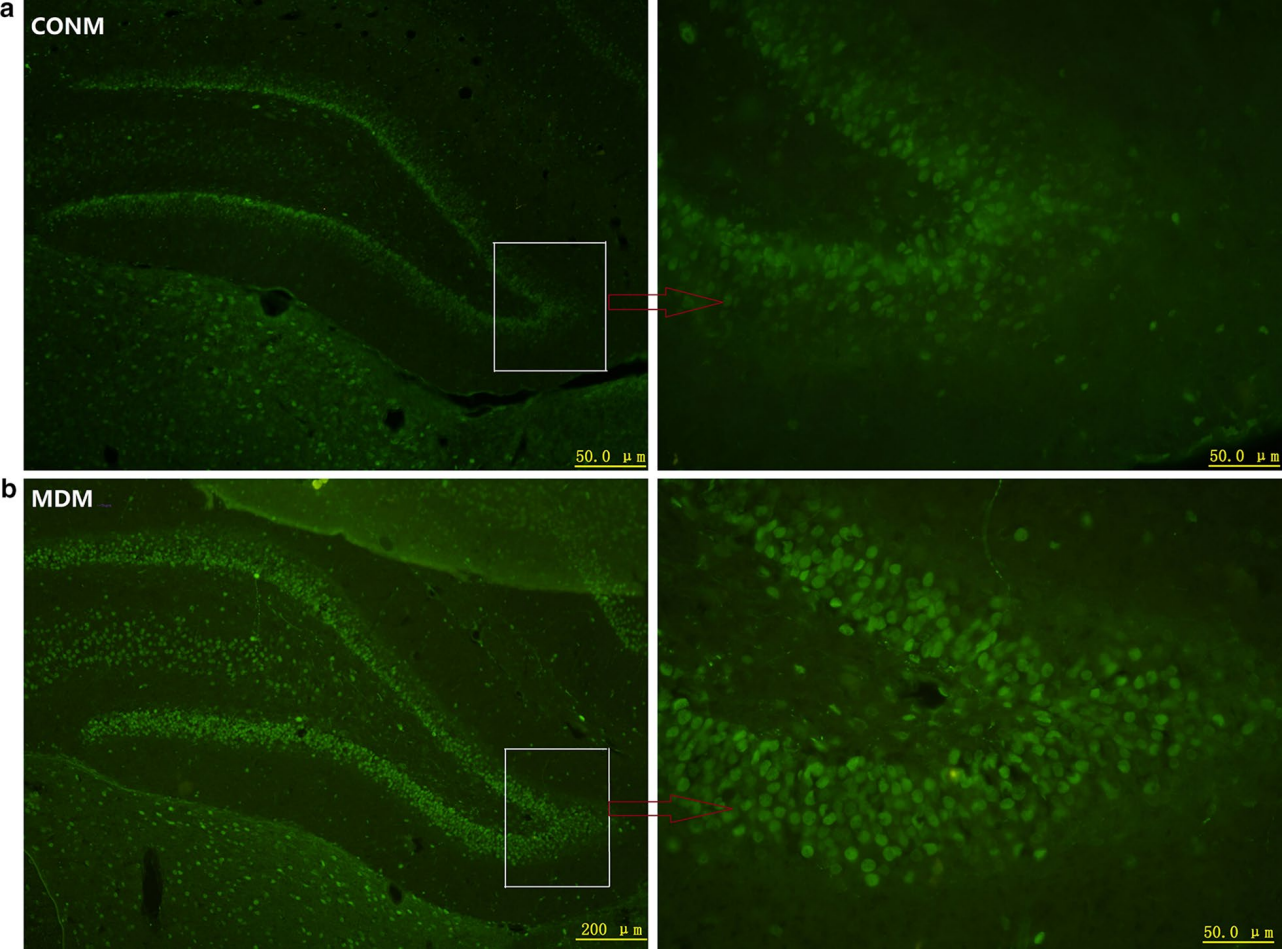
CALR evident in the dentate gyrus of the hippocampus. **a** CALR expression in control male rats; **b** CALR expression in MD male rats

**Fig. 2:**
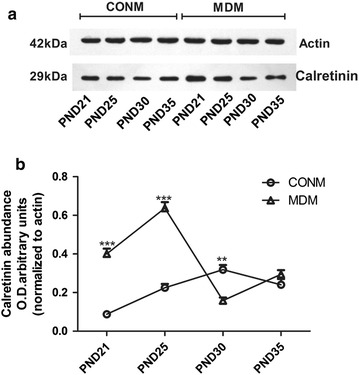
CALR levels in the hippocampus of male rat determined by Western blot (four immunoblots for CALR were analyzed. CALR levels in the (**a**) male rat hippocampi on PND-21, -25, -30, and -35. Quantification is shown in (**b**). *CONM* control males, *MDM* MD-treated males. **p <0.01, ***p <0.001

On PND-21, abundant CALR protein was observed in the dentate gyrus of the hippocampus in both the MD and control groups (Fig. 1). In the male rats of the MD and control groups, the hippocampal CALR levels were at maximum on PND-25 and PND-30, respectively. Two-way ANOVA for CALR level across the timepoints showed significant effects of treatment (F_(1,40)_ = 29.78, *p *< 0.001), time (F_(3,40)_ = 37.28, *p *< 0.001) and interaction (F_(3,40)_ = 15.7, *p *< 0.001). CALR levels in the MD males were much lower than that of the control males on PND-21 and PND-25, but higher on PND-30. However, on PND-35, the CALR levels of the MD and control males were statistically comparable (Fig. 2a, b).

**Fig. 3:**
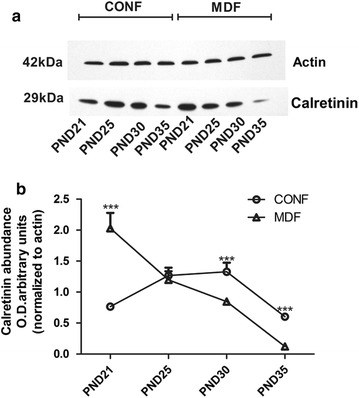
CALR levels in the hippocampus of female rat determined by Western blot (four immunoblots for CALR were analyzed. CALR levels in the (**a**) female rat hippocampi on PND-21, -25, -30, and -35. Quantification is shown in (**b**). *CONF* control females, *MDF* MD-treated females. ***p <0.001

The trends in hippocampal CALR levels described above for the males differed for the females. Two-way ANOVA for CALR level across the timepoints showed significant effects of time (F_(3,40)_ = 163.05, *p *< 0.001) and interaction (F_(3,40)_ = 134.11, *p *< 0.001), but the effect of treatment was not significant (F_(1,40)_ = 2.61, *p *> 0.05). In the female rats of the MD and control groups, the hippocampal CALR levels reached maximum on PND-21 and PND-30, respectively. On PND-25, the CALR levels of the females in the MD group were similar to that of the females in the controls. However, on PND-30 and PND-35, these levels in the MD females were much lower than in the control females (Fig. 3a, b).

### Hippocampal CALB from PND-21 to PND-35

**Fig. 4:**
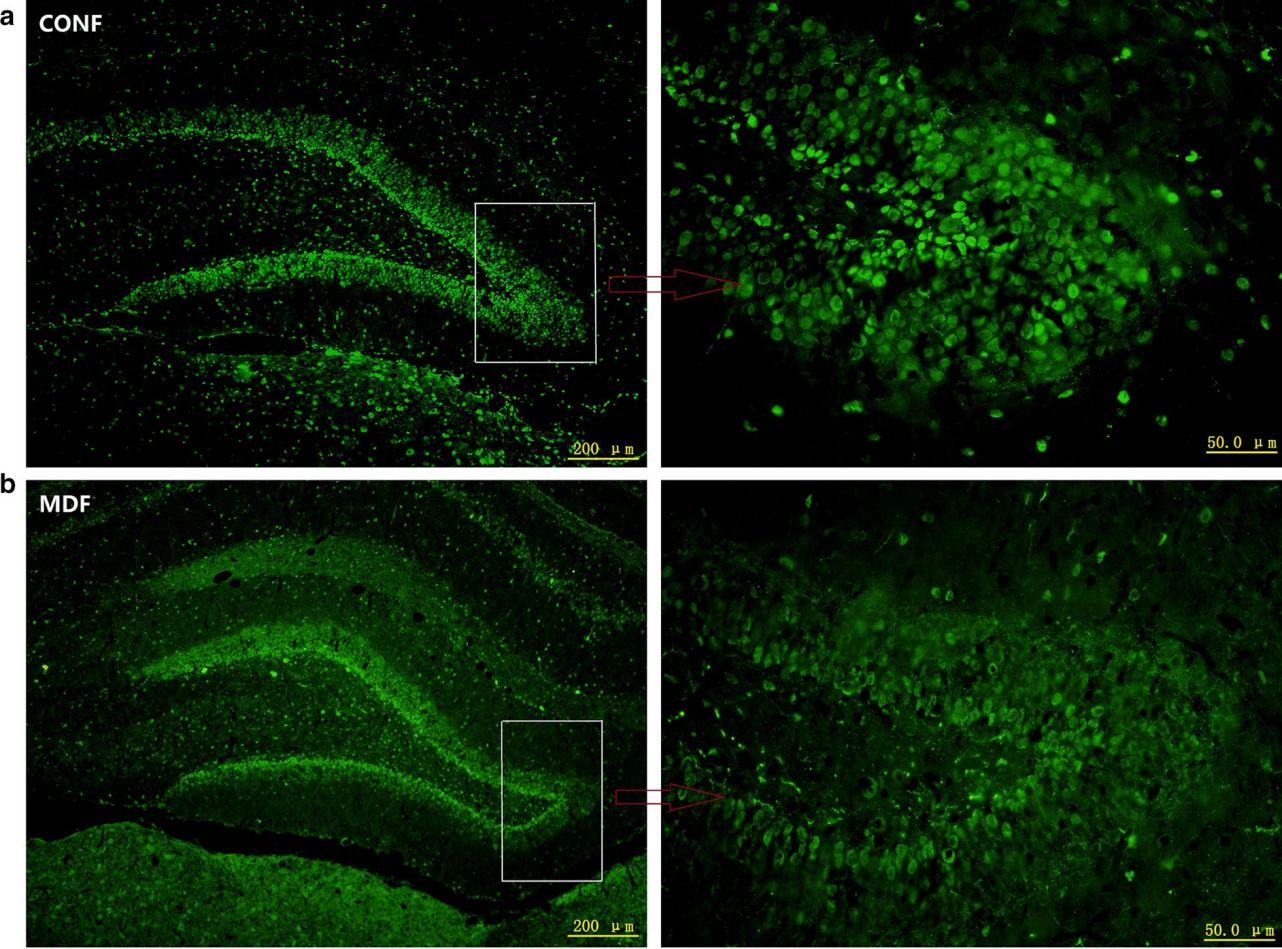
CALB in the dentate gyrus of the hippocampus. **a** CALB expression in control female rats; **b** CALB expression in MD female rats

**Fig. 5:**
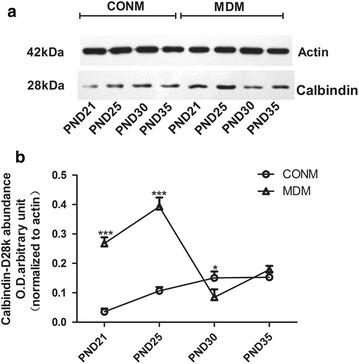
CALB levels in the hippocampus of male rat determined by Western blot (four immunoblots for CALB were analyzed). CALB levels in the (**a**) male rat hippocampi on PND-21, -25, -30, and -35. Quantification is shown in (**b**). *CONM* control males, *MDM* MD-treated males. *p <0.05, ***p <0.001

**Fig. 6:**
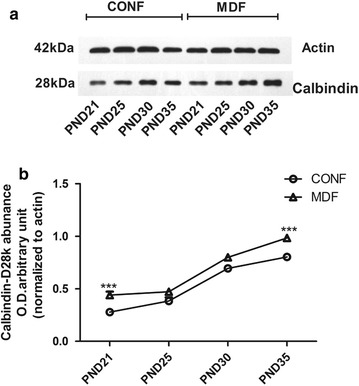
CALB levels in the hippocampus of female rat determined by Western blot (four immunoblots for CALB were analyzed). CALB levels in the (**a**) female rat hippocampi on PND-21, -25, -30, and -35. Quantification is shown in (**b**). *CONF* control females, *MDF* MD-treated females. ***p <0.001

Similar to CALR protein, CALB protein in the dentate gyrus of the hippocampus was also abundant (Fig. 4). In the male rats, two-way ANOVA for CALR level across the timepoints showed significant effects of time (F_(3,40)_ = 88.18, *p *< 0.001), treatment (F_(1,40)_ = 400.49, *p *< 0.001) and interaction (F_(3,40)_ = 194.81, *p *< 0.001). Like the CALR protein, the CALB levels in the MD males were much higher than that of the control males on PND-21 and PND-25, and lower on PND-30. However, on PND-35, the difference in the CALB levels of the males of the MD and control 2 groups was not significant (Fig. 5a, b). The CALB levels of the females differed from the CALB levels of the males. Two-way ANOVA for CALR level across the timepoints showed significant effects of time (F_(3,40)_ = 1289.56, *p *< 0.001), treatment (F_(1,40)_ = 359.99, *p *< 0.001) and interaction (F_(3,40)_ = 10.06, *p *< 0.001). From PND-21 to PND-35, in the females of both the MD and control groups, the quantity of CALB increased gradually. The level of hippocampal CALB was much higher in the MD females than in the control females on PND-21 and PND-35, but on PND-25 and PND-30 the 2 groups were similar (Fig. 6a, b).

### Exploring behavior: the open-field test

**Fig. 7:**
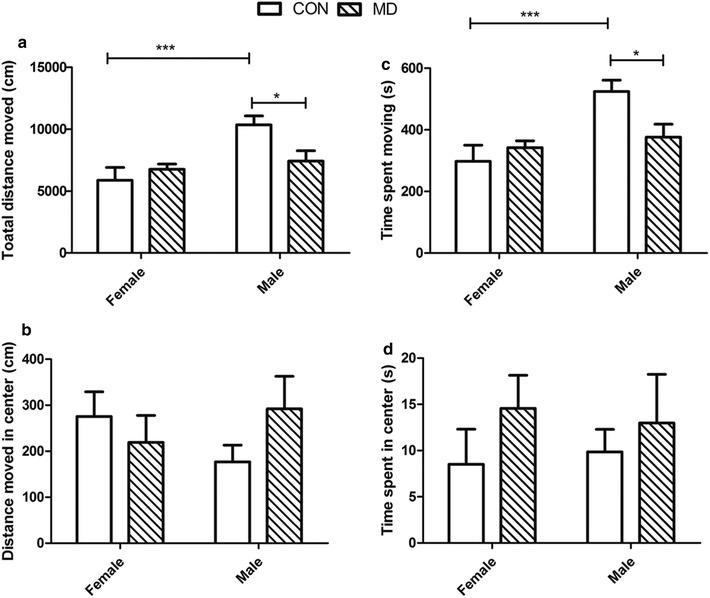
Open field test (six rats from each group were tested). In an open field arena, **a** total distance moved and **b** distance moved in center are considered as an index of reduced exploring behavior, **c** time spent moving and **d** time spent in center are considered as an index of anxiety. *p <0.05, **p <0.01, ***p <0.001

During the open-field test, there were significant differences between the sexes in total distance moved (F_(1,44)_ = 10.66, *p *< 0.01) and time spent moving (F_(1,44)_ = 10.66, *p *< 0.01; Fig. 7). Male rats in the MD group moved a shorter distance (*p *< 0.01) and with less time spent moving (*p *< 0.05), relative to the males of the control group, but the females of the 2 groups were similar for both features (Fig. 5). Neither the sexes nor the groups differed with regard to the distance (sexes: F_(1,44)_ = 0.05, *p *> 0.05; treatment: F_(1,44)_ = 0.28, *p *> 0.05) and time spent moving (sexes: F_(1,44)_ = 0, *p *> 0.05; treatment: F_(1,44)_ = 1.37, *p *> 0.05) in the center of the open field arena.

### Learning and memory: the MWM test

**Fig. 8:**
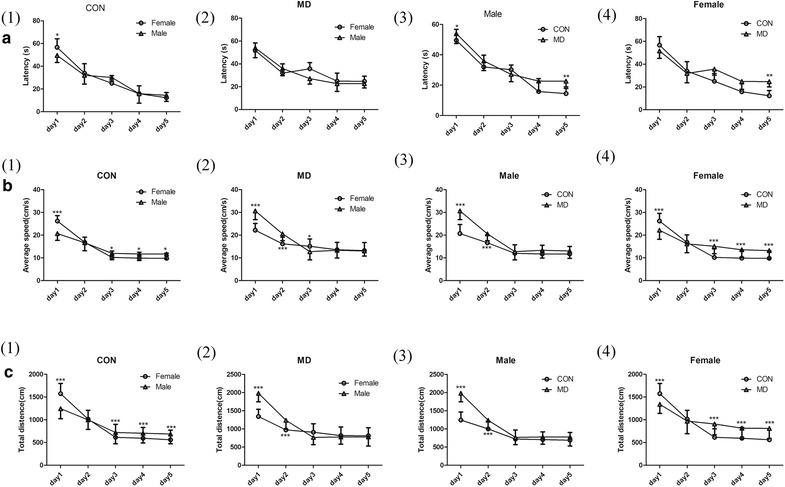
MWM test (eight rats from each trail group were tested). Escape latency interval (the length of time needed to find the hidden platform) for each of the five training days were shown. Longer escape latency interval is considered as reduced learning ability. The latency (**a**), the average speed (**b**) and total distance (**c**). *CON* control rats, *MD* MD-treated rats. *p <0.05, **p <0.01

On PND-25, half of the rats (eight females and eight males from each of the MD and control groups) were randomly chosen for the MWM test. When the analysis was controlled for treatment, two-way ANOVA for latency across the timepoints showed significant effects of time [control group: F_(4,310)_ = 360.65, *p* < 0.001, Fig. 8a(1); MD group: F_(4,310)_ = 223.97, *p *< 0.001, Fig. 8a(2)] and sex by time interaction (control group: F_(4,310)_ = 7.57, *p *< 0.001, Fig. 8a(1); MD group: F_(4,310)_ = 3.25, *p* < 0.001, Fig. 8a(2), but there was no significant effect of sex [control group: F_(1,310)_ = 0.24, *p *> 0.05, Fig. 8a(1); F_(1,310)_ = 3.25, *p *> 0.05, Fig. 8a(2)]. However, when the analysis was controlled for sex, repeated two-way ANOVA for latency across the timepoints showed both significant effects of time [male group: F_(4,310)_ = 316.16, *p *< 0.001, Fig. 8a(3); female group: F_(4,310)_ = 288.75, *p *< 0.001, Fig. 8a(4)], treatment [male group: F_(1,310)_ = 35.98, *p *< 0.001, Fig. 8a(3); female group: F _(1,310)_ = 44.67, *p *< 0.001, Fig. 8a(4)], and treatment by time interaction [male group: F_(4,310)_ = 8.13, *p *< 0.001, Fig. 8a(3); female group: F_(4,310)_ = 22.54, *p *< 0.001, Fig. 8a(4)]. Post hoc analysis with Bonferroni correction showed that though male rats of the MD group swam faster significantly [*p *< 0.001, Fig. 8b(3)], they also swam much longer distance [*p *< 0.001, Fig. 8c(3)], so they still spent more time finding the escape platform than the male rats of the control group on the first day [*p *< 0.05, Fig. 8a(3)]. At the last day, males in MD group also spent more time to find the platform [*p *< 0.01, Fig. 8a(3)], although they had the same average speed [*p *< 0.001, Fig. 8b(3)]. Except for the 2nd day, females rats of the MD group had significantly higher speed than the ones from control group [*p *< 0.001, Fig. 8b(4)], they only had a longer latency escape interval on the last day [*p *< 0.01, Fig. 8a(4)], but on the other days the females of the 2 groups spent similar time to find the platform [*p *> 0.05, Fig. 8a(4)].

### Effect of MD and/or MWM test on CALR and CALB levels on PND-40

**Fig. 9:**
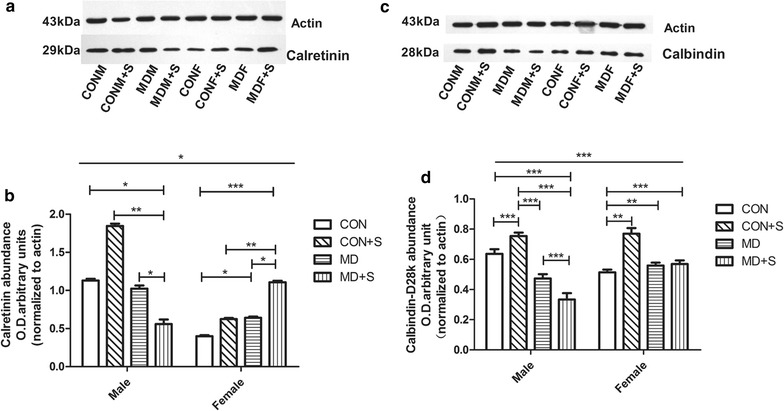
Hippocampal CALR and CALB levels after MWM on PND-40 determined by Western blot. **a**, **c** Representative blots (four immunoblots for each protein were analyzed). **b**, **d** Quantification. *p <0.05, **p <0.01, ***p <0.001. *CONF* control females, *CONF + S* control females who underwent MWM training, *CONM* control males, *CONM + S* control males who underwent MWM training, *MDF* MD-treated females, *MDF + S* MD-treated females who underwent MWM training, *MDM* MD-treated males, *MDM + S* MD-treated males who underwent MWM training

On PND-40, the hippocampal CALR levels of the females and males differed significantly (F_(1,40)_ = 4.53, *p *< 0.05; Fig. 9a, b). Male rats that were exposed to both MD and the MWM test had significantly lower CALR levels compared with the males that were exposed to MD alone (*p *< 0.05) or the MWM test alone (*p *< 0.001). Females that were exposed to MD alone had significantly higher CALR levels compared with females of the control group (*p *< 0.05). Females that were exposed to both MD and the MWM test had significantly higher CALR levels compared with females exposed to MD alone (*p *< 0.05), or only the MWM test (*p *< 0.01).

Similar to the CALR results, the CALB levels were also significantly different between the females and males (F_(1,40)_ = 10.74, *p *< 0.01; Fig. 7c, d). Males that were exposed to the MWM test alone had higher CALB levels than the males from the control group (*p *< 0.001). Males exposed to the MWM test alone had higher CALB levels than the males exposed only to MD (*p *< 0.001). Males exposed to only the MWM test had higher CALB levels than those that were exposed to both MD and the MWM test (*p *< 0.001). Males exposed to both MD and the MWM test had lower CALB levels compared with the males of the control group (*p *< 0.001) and also compared with males exposed only to MD (*p *< 0.001).

Female rats exposed only to the MWM test, exposed only to MD, or exposed to both MD and the MWM test, had significantly higher CALB levels than the females of the control group (*p* < 0.01, < 0.01, and < 0.001, respectively).

## Discussion

In the present study, we investigated the effects of early-life adversity on hippocampal CALR and CALB protein levels and cognitive behaviors, and explored whether these effects were sex-related.

On PND-21, CALR and CALB levels were elevated in the MD group both in male and female rats, which was in accordance with our previous study [32]. On PND 35, CALR and CALB returned to the normal level in male rats exposed to MD, but female rats exposed to MD showed lower CALR level and slightly higher CALB level compared with controls.

In the open field test, we observed that male rats exposed to MD explored less, but female rats were not affected by MD exposure. Our previous study also showed that only male but not female rats exposed to MD spent less time in the open arm in the elevated plus maze test [32]. The decrease in exploration behavior and avoidance of stressful environment were indications of anxiety-like behaviors in rodents [37–40]. The anxiety-like behaviors were reported to be caused by elevated HPA reactivity [41, 42]. High glucocorticoid levels, on the other hand, induce neuronal activation and increased Ca^2+^ influx into neurons [43, 44]. Excessive intracellular Ca^2+^ was neurotoxic, and calcium-binding proteins, CALR and CALB, buffer excessive intracellular Ca^2+^ levels and therefore have an important role maintaining neuronal homeostasis and survival [43, 45, 46]. In our previous study, we observed that female rats had 4–5-fold higher CALB levels in the hippocampus compared with males [32], and possibly a stronger ability to buffer stress induced by excessive Ca^2+^ influx into neurons. This may explain why the female rats were more resistant to MD stress. Although the CALR and CALB levels were dramatically increased by 3–5-fold in male MD rats during PND-21 and PND-25, the levels probably could still not compensate for the excessive intracellular Ca^2+^ that is harmful to brain homeostasis.

Interestingly, both male and female rats exposed to MD showed impaired spatial memory on the last day of the Morris water maze test. We observed that both male and female rats that were not exposed to MD stress had elevated hippocampal levels of CALR and CALB after Morris water maze test, and this was possibly an indication of increased neuronal activity and brain plasticity after spatial learning. However, in the MD group, both CALR and CALB levels were lower in male rats after MWM test, which might cause their impairment in spatial learning. Unlike male rats, higher CALR level and comparable CALB level were observed in female MD group compared with control group, indicating a different mechanism underlying the spatial learning impairment in female MD rats.

CALR was reported to have important role in neurogenesis and neuroprotection [47, 48] but did not affect the spatial learning in rodents [35, 36], while CALB was reported to affect both learning and neuroprotection [49–52]. In the present study, it was possible that the failure to increase the CALB level after spatial learning limited the brain plasticity and caused the impairment in learning in female MD rats. Although female rats developed a mechanism to counteract MD stress and did not show abnormal behaviors under non-stressful conditions, there may still be minor changes in their brain plasticity when exposed to MD stress. The cognition impairment caused by MD in female rats could probably only be visualized under challenged conditions, such as spatial learning and the stressful environment caused by MWM test. We believe that sex hormones had an important role in regulating calcium binding protein responses differently in male and female rats, but the detailed mechanisms remain to be investigated.

In the current study, we demonstrated that male rats were more vulnerable to MD stress than female rats, and showed decreased explorative behavior and spatial learning impairment after MD exposure. Most importantly, we found that female rats, although resistant to MD stress to a certain level, demonstrated also spatial learning impairment after exposure to MD stress. The spatial learning impairment in female MD rats may be caused by limited brain plasticity, especially lack of involvement of CALB protein, during the MWM test. This study provides evidence of a sex difference in neurodevelopmental disorders and suggests the involvement of calcium-binding proteins in developmental abnormalities caused by early-life adversity.

## Abbreviations

MD: maternal deprivation
CALR: calretinin
CALB: calbindin-D28k
PND: postnatal day
MWM: Morris water maze
CNS: central nervous system
ADHD: attention-deficit/hyperactive disorder
ASD: autism spectrum disorder
